# Prenatal diagnosis of clinodactyly and its association with genetic syndromes: A case report

**DOI:** 10.1016/j.crwh.2024.e00674

**Published:** 2024-12-06

**Authors:** Themistoklis Loukopoulos, Athanasios Zikopoulos, Nikolaos Vlassis, Emmanouil Manolakos, Sotirios Sotiriou, Anastasia Vatopoulou, Fani Gkrozou, Anastasios Potiris, Sofoklis Stavros, Charikleia Skentou

**Affiliations:** a Department of Obstetrics and Gynecology, University Hospital of Ioannina, Ioannina, Greece; b Department of Obstetrics and Gynecology, Royal Devon and Exeter Hospital, Barrack Rd, Exeter EX2 5DW, UK; c Obstetrician & Gynecologist, Private Practice, Lamia, Greece; d Access To Genome-ATG, Clinical Laboratory Genetics, Athens, Greece; e Department of Embryology, Faculty of Medicine, University of Thessaly, Larissa, Greece; f Department of Obstetrics and Gynecology, University Hospital of Attikon Athens, Greece

**Keywords:** Clinodactyly, Prenatal ultrasound, Genetic counseling, Molecular karyotyping

## Abstract

A curvature of a finger that bends inwards relative to the other fingers is a common observation during prenatal screening. When the angulation exceeds 10 degrees, it is known as “clinodactyly” and could suggest a variety of underlying issues. Even though it usually remains unnoticed during pregnancy, it may be a sign of serious fetal disease. We report the case of a fetus diagnosed with clinodactyly of the thumb accompanied by tachycardia, abnormal levels of maternal hormones in the first trimester and increased impedance to flow in the uterine arteries. Although non-invasive prenatal testing was normal, amniocentesis was carried out and two deviations at chromosome 20 were identified through molecular karyotyping. Our report aims to raise clinical suspicion regarding the potential association between genetic abnormalities and clinodactyly. A careful clinical and genetic consultation is required in order to achieve the most favorable outcome for both mother and child.

## Highlights

• Fetal tachycardia in the first trimester is a key risk factor for chromosomal abnormalities.
• Clinodactyly on ultrasound may prompt genetic testing for potential underlying disorders.
• Clinodactyly combined with other abnormalities requires a thorough multidisciplinary approach.
• Early identification and counseling are vital for informed prenatal management.

## 1 Introduction

Clinodactyly, defined as an aberrant curvature of more than 10 degrees in one or more fingers or toes in a coronal plane, is a challenging clinical situation in prenatal diagnosis that poses problems for both medical professionals and families [1]. This congenital defect, which can be identified prenatally, may be a sign of underlying genetic disorders, although it is usually benign [2]. The prevalence of clinodactyly varies across populations, with estimates ranging from 1 % to 19.5 % in the general population [1,3,4]. The fifth finger's middle phalanx is most usually affected but several studies have also reported involvement of the proximal phalanx of the index finger and thumb [5].

While clinodactyly may occur independently, its diagnosis necessitates careful examination to rule out associated genetic disorders. Notably, clinodactyly is present in up to 25 % of Down syndrome cases; however, it has been also linked to a variety of genetic abnormalities and syndromes, including Turner, Klinefelter and Rubinstein-Taybi syndromes, Cenani-Lenz syndactyly, Fanconi anemia and a wide range of chromosomal defects and monogenic disorders1.

As prenatal screening techniques and genetic testing advance, comprehending the importance of clinodactyly during pregnancy becomes increasingly important for accurate diagnosis, prognosis, and informed decision-making by new parents. As a result, early and accurate identification of the disorder through prenatal ultrasound is of great significance to determine possible underlying genetic associations.

Here we report a case of prenatal identification of clinodactyly linked to a genetic disorder, in order to raise clinical suspicion of the association between this common sonographic finding and the possible underlying genetic disorders.

## 2 Case Presentation

A 25-year-old woman (G1P0) was referred for prenatal diagnosis because she had a high risk for Patau's syndrome (trisomy 13). Her medical, social and family history was unremarkable.

Fetal ultrasound was performed at 12 2/7 weeks of gestation and revealed normal anatomy and a nuchal translucency measured at 1.30 mm; however, the fetus had persistent tachycardia (181 bpm). The other first-trimester ultrasound markers, including nasal bone, ductus venosus and tricuspid valve Doppler, were in normal ranges. The biochemistry results were abnormal (see Table 1) [6,7]. Given these findings, the chance of Patau's syndrome increased to 1/2322, whereas the odds of Down syndrome and Edward syndrome decreased significantly.

**Table 1:** The median multiple of the median (MoM) of maternal serum free beta-human chorionic gonadotrophin (b-hCG), pregnancy associated plasma protein A (PAPP-A) and placental growth factor (PlGF) at 12 2/7 weeks of gestation was significantly decreased.

	Value
β-hCG	14.99 IU/l (0.370 MoM)
PAPP-A	0.780 IU/l (0.450 MoM)
PlGF	8000 pg/ml (0.226 MoM)

Additionally, an increased mean pulsativity index (PI) of the uterine arteries was also observed measured at 2.730 (1.523 MoM). Τhe risk for pre-eclampsia before 37 weeks of gestation was calculated at 1/164 and the risk of fetal growth restriction was estimated to be 1/37. Accordingly, low-dose aspirin (160 mg daily) was prescribed.

The couple opted for non-invasive prenatal testing due to the increased probability of Patau syndrome. The result indicated a low-risk pregnancy for common trisomies with a fetal DNA fragment of 6,5 %.

**Fig. 1:**
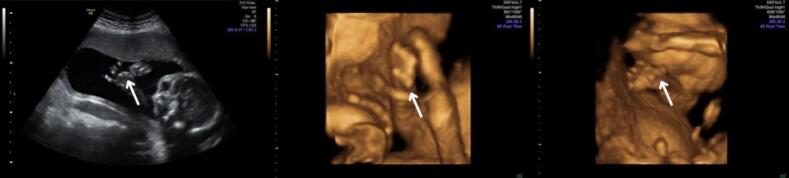
Two-dimensional and three-dimensional ultrasound images showing the curvature of the thumb indicating clinodactyly.

During the next weeks, the patient had close monitoring with ultrasound scans every 3 weeks and no further abnormality was observed besides tachycardia, which l persisted. Nevertheless, anomaly scan at 21 3/7 weeks detected a persistently curved thumb of the fetus' right hand (Fig. 1).

Ultrasound was repeated 2 days later and the diagnosis of clinodactyly was established. Given the fact that clinodactyly can often occur as a part of a genetic syndrome, in combination with tachycardia, the woman was referred to a specialized pediatric cardiologist. Fetal heart ultrasound revealed two perimembranous ventricular septal defects.

**Fig. 2:**
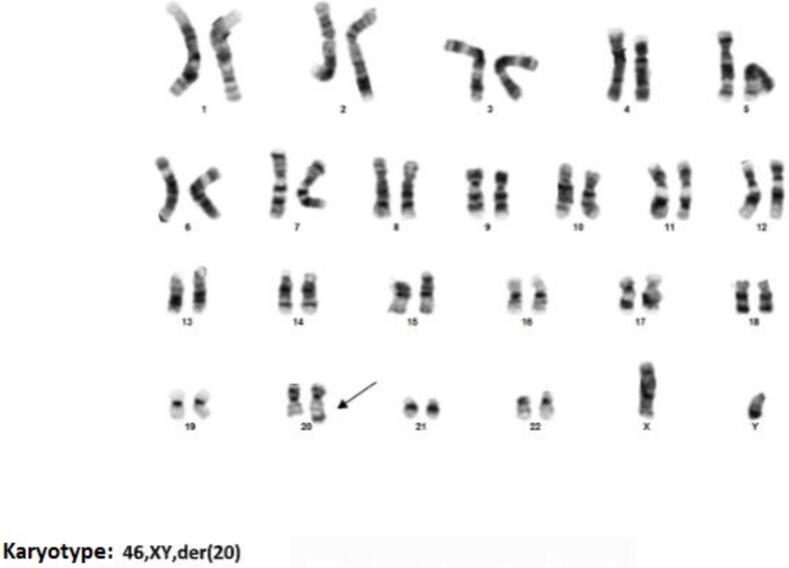
GTG banding of metaphase chromosomes from the amniotic fluid.

At 22 weeks of gestation amniocentesis was performed uneventfully due to maternal anxiety and ultrasound findings and array-CGH was carried out using DNA derived from the amniotic sample. A 24 Mb duplication at chromosome 20, spanning 20p13 to 20p11.21 (positions 60,747,545 to 24,031,374) and a 526 kb deletion in 20q13.33 (positions 62,382,404-62,908,674) were identified (Fig. 2).

**Fig. 3:**
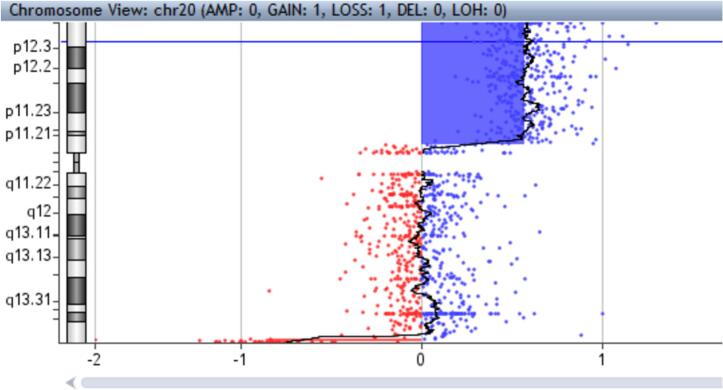
Graph of the array-CGH of chromosome 20.

After cell culture, chromosomes prepared from amniotic fluid revealed in GTG banding (300–400 bands), a derivate chromosome 20 being present in two independent cultures (karyotype: mos 46,XY, der(20) (Fig. 3).

Parental peripheral blood samples analyses revealed a normal paternal karyotype but a pericentric inversion of one chromosome 20 was observed in the maternal sample. The likely breakpoints were defined as 20p11.1 and 20q13.3.

Pericentric inversions of chromosomes are generally not associated with pathological clinical features but they have been linked to reproductive issues as well as the possibility of having a child with unbalanced genetic material. Depending on the type of the chromosomal abnormalities, this can result in severe developmental and health problems.

During genetic counseling, thorough explanations of the significance of the detected chromosomal alterations were provided, including the potential risks of congenital defects or developmental delays. The couple also received information regarding the phenotype associated with the karyotype 46, XY, der(20), emphasizing the uncertainties and potential outcomes, due to unbalanced genetic material.

After a detailed discussion and consideration of the information provided, the parents decided to terminate the pregnancy, which was done uneventfully at 26 weeks.

## 3 Discussion

Clinodactyly is frequently discovered accidentally during prenatal ultrasound imaging. The disorder may appear as a single defect or as a component of a larger syndromic pattern, particularly when chromosomal abnormalities such Down syndrome (trisomy 21) and other genetic disorders are present [2]. Ultrasound is an important tool for detecting limb malformations during pregnancy, such as clinodactyly. According to Ermito et al., comprehensive fetal ultrasonography is critical for proper diagnosis since limb anomalies can be an early sign of more complicated disorders, such as chromosomal abnormalities and genetic syndromes [8].

Clinodactyly is linked to chromosomal abnormalities, mainly trisomy 21, which is observed in up to 25 % of cases [1]. However, when is the only ultrasound finding, clinodactyly has low prognostic relevance and does not usually suggest a poor prognosis. According to Paladini et al., isolated abnormalities of the upper extremity, such as clinodactyly, have a better prognosis than multiple deformities, which indicate a genetic syndrome [9]. The discovery of clinodactyly should motivate a thorough ultrasound scan for other markers and anatomical defects, as well as genetic testing. In particular, essential ultrasound markers that should be evaluated include: nuchal transiency, since increasing thickness may suggest chromosomal abnormalities, particularly Down syndrome; absent or hypoplastic nasal bone, which is also frequently seen in Down's syndrome; echogenic intracardiac focus, which can often be observed in aneuploid fetuses; and a shortened femur or humerus, which may indicate a growth abnormality.

3D ultrasound can be an effective tool to assess fetal limb morphology, providing clearer visualization of clinodactyly and other limb anomalies, as illustrated in Fig. 1.

Even though clinodactyly on its own is relatively common, when combined with other indicators, such as the ones mentioned above, it increases the possibility that underlying chromosomal problems are present [10].

More syndromes like Turner syndrome, Klinefelter syndrome, and Apert syndrome**,** can be present, with similar limb deformities, so it is crucial to include them in differential diagnosis when clinodactyly is identified [1,11,12]. Furthermore, a detailed family history and parents' clinical assessment should be evaluated, given that familial patterns of clinodactyly may indicate a genetic predisposition or related syndromes.

## 4 Conclusion

In summary, this case highlights the significance of detecting clinodactyly at prenatal ultrasound. In addition, it emphasizes the critical responsibility of obstetricians in counseling pregnant women about the significance of this seemingly innocent deviation and its possible associations with fetal conditions.

Given the potential severity of the genetic problems associated with clinodactyly, medical professionals need to provide thorough prenatal counseling, offering all available details about potential genetic connections and relevant diagnostic procedures. Molecular karyotyping, as in this case, can help identify chromosomal anomalies that coexist with clinodactyly, allowing for more informed prenatal management. Clinicians should be aware of key ultrasound indicators and consider using 3D imaging to improve diagnostic accuracy. Moreover, a multidisciplinary approach incorporating neonatologists as well as maternal-fetal medicine specialists, clinical geneticists, sonographers, and genetic counsellors, may be beneficial in addressing neonatal management of clinodactyly and any associated conditions. Collaborative efforts are required to give complete genetic counseling, ensuring the best outcomes for both parents and child.
